# Noseband Fit: Measurements and Perceptions of Canadian Equestrians

**DOI:** 10.3390/ani12192685

**Published:** 2022-10-06

**Authors:** Katrina Merkies, Caleigh Copelin, Nicolas Small, Joelene Young

**Affiliations:** 1 Department of Animal Bioscience, University of Guelph, Guelph, ON N1G 2W1, Canada; 2 Campbell Centre for the Study of Animal Reproduction, University of Guelph, Guelph, ON N1G 2W1, Canada

**Keywords:** taper gauge, competition, ethical training, equine welfare, competition rules

## Abstract

**Simple Summary:**

There is a concern for the welfare of competition horses and the issues presented by incorrect noseband fit. Nineteen volunteer Stewards used a noseband taper gauge to measure the noseband fit of 551 competition horses during the 2021 season at a variety of Canadian equestrian events. Over 70% of competition horse nosebands measured met the two-finger rule. Stewards agreed that a standardized measure was useful across disciplines although they did not agree that it should be mandatory. Surveys were collected from 27 Stewards and 1528 members of Equestrian Canada to gather perceptions and opinions on noseband use and fit. The most common type of noseband was a cavesson and respondents indicated they used a noseband mostly because it was expected or for safety and control reasons. Riders expressed a desire for the option to not wear a noseband in competition. Professional riders were more distrustful of using a taper gauge to measure noseband fit and did not feel overtightened nosebands were as much of an issue as amateur riders. To advance equestrian practice, more education is needed on the reasons for noseband measurements and appropriate fit.

**Abstract:**

Recent concerns regarding horse welfare during competition has highlighted the occurrence of overtightened nosebands on competition horses. Current rules are often vague—e.g., “nosebands may never be so tightly fixed as to harm the horse.” To investigate the need and acceptance prior to any rule changes Equestrian Canada (EC) launched a pilot noseband measuring project. Nineteen officiating stewards measured noseband fit using the ISES taper gauge (TG) at 32 equestrian events of various disciplines in 2021. Additionally, stakeholder surveys collected data from 1528 EC members and 27 stewards regarding opinions and perceptions on noseband use, fit, measurement and rules. Descriptive and qualitative statistics along with Pearson chi-squared examined relationships between specific variables. Of the 551 horses tested with the TG, 71% passed the 1.5 cm (two-fingers) measurement and an additional 19% passed the 1 cm (one-finger) measurement. Stewards unanimously agreed that overtightened nosebands present a welfare issue although 63% believed this to represent only a small subset of riders. While 60% of stewards believed the current rules were sufficient, 40% did not. Despite the fact that 84% of stewards believe there should be a standardized fit across disciplines, 52% felt the use of the TG should be at their discretion. The top three reasons riders indicated for using nosebands were discipline expectation (41%), requirement for competition (39%) or for control/safety (32%). Open comments referred to an option to not wear a noseband in competition. Professional riders believed overtightened nosebands were less of a welfare issue than amateur riders (76% vs. 88% respectively; *p* < 0.025) and correspondingly did not feel the TG was a fair method (44% vs. 68% respectively; *p* < 0.001). Slightly more than half of the respondents (51.5%) believed that measuring noseband fit on the frontal nasal plane was the appropriate location. To advance equestrian practice, more education is needed to inform stakeholders of the reasons for noseband measurements and appropriate fit.

## 1. Introduction

Nosebands have been depicted as part of a horse’s bridle in early depictions of ridden horses dating to almost 4000 years ago [1]. Noseband design varies from a simple cavesson fitted just below the facial crest to those that encircle the horse’s muzzle in front of the bit with many variations. The purpose of the noseband is to provide more control to the rider by preventing the horse from opening their mouth and thus sensitizing them to bit pressure [2]. When used correctly a noseband works through negative reinforcement to teach the horse to properly carry the bit—pressure under the noseband increases when the horse opens their mouth in an attempt to evade the bit and the pressure is released when the horse closes their mouth [3,4]. Beginners to horseback riding are taught the generally accepted methods for tacking up a horse including being able to slide two fingers under the noseband as a gauge for the correct fit [2]. Efforts to increase rider control and prevent evasion of the bit by the horse have led to nosebands being fastened more tightly than the usual two fingers [2,5,6,7]. While an increase in rider control over a large and potentially dangerous animal has merits in improving rider safety [8], consideration must also be given to the display of conflict behaviors in the horse as a result of incorrectly fitted nosebands, which paradoxically may jeopardize rider safety [9].

Nosebands tightened to the point of having no space between noseband and nasal plane resulted in a decrease in normal oral behaviors such as yawning, licking and swallowing [10] followed by a post-inhibitory rebound of these behaviors once the noseband was removed. Heart rate and eye temperature also increased in horses whose nosebands were overtight [10,11] suggesting a stressful event. A study of over 3000 Danish competition horses found that tighter nosebands were associated with the prevalence of lesions at the corner of the lips where the bit sits [12]. Horses resistant or evasive to bridling may perceive an association between wearing tack and pain [13].

The facial artery and vein as well as the salivary glands and branches of the facial nerves run in the area where a standard cavesson noseband sits beneath the facial crest on the horse’s face [14]. These delicate tissues may be damaged if frequently exposed to high pressures from nosebands. Studies have found that the pressures of an overtight noseband can be compared to or even exceed those of a tourniquet used in humans [15,16]. Crank nosebands, those that employ a leverage action to enable tighter fastening, resulted in peak pressures on the horse’s face of 200–400 mm Hg [5] and cooler facial skin [11] supporting the idea that tight nosebands impair blood flow in the face. Nerve damage in humans from tourniquet use can occur with pressures of 50 mm Hg [17]. Several studies have observed bony changes at the site of the noseband and the possibility that nosebands may be associated with bone remodeling should not be overlooked [18,19]. This is a major area of concern for equine welfare.

Horse welfare is a high priority for regulating bodies such as the Federation Equestre Internationale (FEI) and although rules have been put into place specifically regarding nosebands, they remain quite vague. For example, the FEI Dressage rulebook states that nosebands permitted in competition “may never be so tightly fixed that it causes harm to the horse” [20]. In many types of equestrian competition, particularly dressage, opening the mouth or other conflict behaviors related to bit pressure are frowned upon by judges, as they indicate a lack of submission and harmony. However, “submission” scores in dressage are subject to the most variability of all marks [21] and may be influenced by various practices of noseband use and fit.

Equestrian Canada (EC), the national regulating body of equestrian sports in Canada, has more comprehensive noseband regulations in their Dressage and Para-Dressage Rules:

“A horse’s noseband must not be over tightened. It must be possible to place at least one finger between the horse’s cheek and the noseband. Nosebands must never be used in such a way that they interfere with the horse’s breathing. If it is deemed to be tight enough to cause pain or discomfort when presented at the tack check or seen in the warm-up or anywhere on the event location, the steward or his/her appointee will consult with the judge and ask the athlete to loosen it before riding any further tests. Failure to comply with this request will result in disqualification” [22].

While some noseband-based protections are in place in national and international rulebooks, the wording remains ambiguous and difficult to enforce. Questions prevail such as: how are parameters such as harm and injury defined? How can one tell if their noseband is tight enough to cause pain, or risk injury or impede airflow? What is the space of one finger?

**Figure 1:**
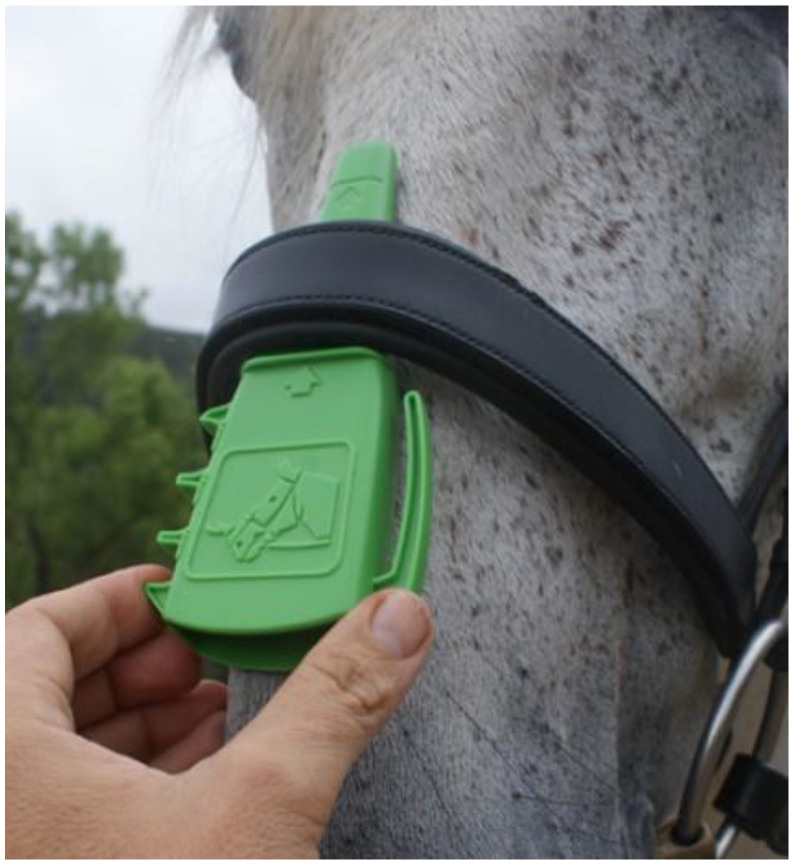
The ISES Noseband Taper Gauge being used to measure a noseband on the frontal nasal plane of the horse—the raised stop mark indicates how far the device should be able to be inserted under the noseband to indicate proper two-finger (1.5 cm) fit. Photo courtesy of ISES.

The industry accepted measure for an appropriately fitted noseband is a measurement of a two-finger space between the noseband and the horse’s face [11,19]. However, finger size and location of the measurement is very subjective. To address this, a noseband taper gauge (TG) was designed to create a more objective form of measurement of the space between a noseband and the horse’s face [23]. The TG manufactured by the International Society for Equitation Science (ISES) has standardized reference points for a measurement of a one-finger space (1 cm from the face) or a two-finger space (1.5 cm from the face) and is designed to be inserted between the noseband and the frontal plane of the nose (i.e., the flat part of the horse’s face; Figure 1). This location is recommended over measuring under the chin or on the side of the face as both of those locations have soft tissues that may be compressed by the gauge, resulting in an inaccurate measurement [23].

To investigate noseband fit on horses in various disciplines and at various levels of competition and to understand how members of the Canadian equine industry feel about welfare issues related to noseband fit and the use of a standardized noseband measurement tool at competitions, Equestrian Canada (EC) launched the Noseband Measuring Pilot Project. The project was two-fold with 19 licensed Stewards gathering noseband measurements from 551 horses representing seven different equestrian disciplines across the 2021 show season, followed by surveys completed by 1528 EC members to investigate practices and opinions on noseband fit. The results of this explorative study can help to advance equestrian practice by informing regulatory bodies on the current state of the industry and best directions to take for embracing continued improvements to equine welfare and quality of life for competition horses.

## 2. Materials and Methods

### 2.1. Noseband Measurements at Equestrian Competitions

Prior to the 2021 outdoor competition season, EC distributed training materials to 19 Stewards who volunteered to take part in the noseband measuring pilot project. In 2021, 106 Stewards were registered with EC, thus this group of volunteer Stewards represented just under 18% of stewards in Canada. Steward qualifications ranged from Learner to FEI Level 3. Stewards were licensed for dressage, para-dressage, jumping, eventing and vaulting disciplines, and General Stewards also officiated at hunter, driving and breed shows. Stewards attended competitions in five different Canadian provinces (Alberta, British Columbia, Nova Scotia, Ontario and Quebec).

Training material included guidelines on proper use of the ISES noseband taper gauge (TG). An acceptable measurement was defined as “When the gauge can be inserted without force up to the raised stop, a spacing of at least 1.5 cm is achieved, or ‘two fingers”. Stewards were instructed to insert the gauge from below the noseband (as recommended by ISES) to allow the gauge to slip out should a horse react negatively to the measurement process.

The number of horses measured per show was left up to each Pilot Steward’s discretion and rider participation was voluntary. Rider consent was obtained verbally to protect the anonymity of riders. Stewards were instructed to take measurements in low-stress areas such as when a competitor was at their stall or entering the warm-up ring. All measurements were taken with riders dismounted. Pilot Stewards approached the horse and introduced the gauge slowly. If a horse became reactive, Pilot Stewards were instructed to stop the measurement. To reduce biosecurity risks, Stewards disinfected NTGs with sanitizing wipes and were recommended to wait at least three minutes between measurements after sanitizing.

For each horse, the discipline, reason for selecting the horse, type of noseband and noseband measurement were recorded on mobile tablets using Alchemer (formerly Survey Gizmo, Louisville, CO, USA) online survey software. Pilot Stewards also recorded their perception of how comfortable the horse was with the TG measurement on a scale of 0–10 with 0 being highly avoidant and 10 being very comfortable.

Following the conclusion of the show season, the Pilot Stewards were asked using an online survey hosted on Alchemer about the number of events they officiated, the discipline and level of the events. They were asked about the number of measurements taken and where best to take them, and were able to offer their opinions on current EC rules and standardized measurements for noseband fit through open comment boxes. A total of 16 of the 19 Pilot Stewards completed this survey. The full list of Pilot Steward survey questions is available in Supplement S1.

Additionally, licensed Stewards who were not part of the noseband measuring project were invited to offer their thoughts on rules regarding noseband fit and welfare issues concerning overtight nosebands using an online survey hosted on Alchemer. This survey received responses from 11 Stewards. The full list of Steward survey questions is available in Supplement S2.

All data from the competition measurements and Steward surveys was provided in aggregate form only, thus only descriptive statistics and qualitative analyses using NVivo (v1.6.1, QSR International, Burlington, MA, USA) are presented. Open-ended comments were classified into broad themes for qualitative analysis by three independent researchers who then came to a consensus on the themes.

### 2.2. EC Member Surveys

Equestrian Canada invited all their members to complete a survey either through direct email or by providing a QR code to those competitors who had their horse’s noseband measured. The survey was available in English or French. In 2021, there were 13,668 members registered with EC. The survey received 1528 responses, representing just over 11% of EC members.

The online survey was hosted on Alchemer and recorded the respondent’s discipline(s) and role(s) within the industry. Respondents indicated their reason(s) for using a noseband on their horse and for noseband use at competitions. The survey then asked the respondent’s opinions on overtight nosebands as a welfare issue, the use of the TG as a standardized unit of measurement, what they considered to be appropriate noseband fit, location and method of measurement, and potential rule changes centered on equine welfare. An open-ended comment box was also provided. The full list of Member survey questions is available in Supplement S3.

Raw data was exported to an Excel spreadsheet for analysis. Descriptive and statistical analyses were performed in SPSS (v28.0.1.1, IBM Statistics, Armonk, NY, USA). Pearson chi-squared compared relationships between demographic variables of the respondents and their responses for the questions pertaining to reasons for wearing nosebands, fit and measurement of nosebands and whether they believe noseband fit is a welfare issue. Open-ended comments submitted in French were translated to English and combined with all the English responses whereupon they were classified into broad themes for qualitative analysis using NVivo by three independent researchers who then came to a consensus on the themes.

## 3. Results

### 3.1. Noseband Measurements at Competitions

#### 3.1.1. Steward Survey Quantitative Results

During the 2021 Canadian show season, Pilot Stewards attended 32 competitions and officiated at an average of five events each. All levels of competition from schooling shows to FEI-level international events were covered with Hunter/Jumper events being the most frequent (*n* = 20) followed by dressage events (*n* = 8). Pilot Stewards recorded an average of 10–30 noseband measurements at each event for a total of 551 measurements. The majority of noseband measurements were performed on dressage horses (52.3%, *n* = 288) followed by hunter/jumper (22.3%, *n* = 123) and eventing (11.3%, *n* = 62) horses. Breed sports, driving and vaulting made up 13 measurements (2.4%) with the remaining 65 (11.7%) measurements unspecified.

Competitors either volunteered for the measurement (55.4%, *n* = 305) or were randomly selected (43.0%, *n* = 237). The remaining nine (1.6%) competitors were selected because their horses had shown signs of pain or distress or had nosebands that looked visibly tight (two and seven competitors, respectively).

**Figure 2:**
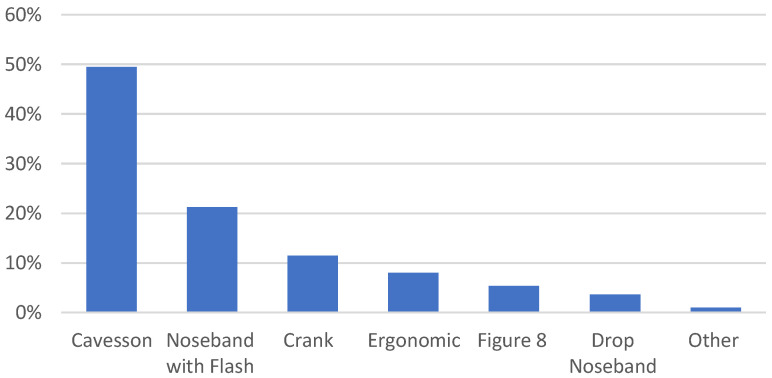
The percentage of different types of nosebands recorded by Pilot Stewards during the 2021 Canadian competition season on a total of 551 horses representing six different equestrian sports.

The most common type of noseband across disciplines was a standard cavesson followed by a noseband with a flash attachment. Crank nosebands, either with or without a flash, constituted 11.4% (63) while various types of nosebands featuring ergonomic designs (e.g., Micklem, Schockemöhle) made up 8% (44). “Other” nosebands included hackamores and crescent nosebands (Figure 2; see Supplement S4 for descriptions of noseband types). Almost half of the measured nosebands (46.5%, *n* = 256) contained additional padding.

**Figure 3:**
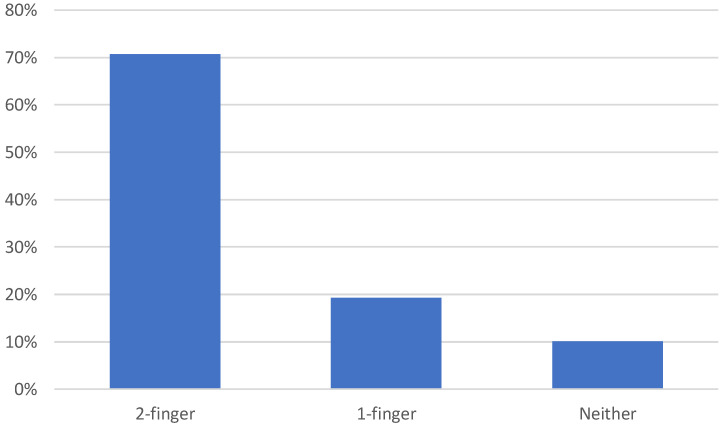
The percentage of horses (*n* = 546) meeting the 2-finger, 1-finger or neither noseband measurement when measured by Pilot Stewards using the ISES taper gauge at Canadian equestrian competitions in 2021.

A large majority of the horses passed the two-finger measurement using the ISES noseband taper gauge. Of those horses who did not pass the two-finger measurement, 19.2% (106) passed the one-finger measurement, with 10.0% (5) of horses having nosebands that were too tight to fit the taper gauge (Figure 3). Six of the 551 horses were unable to be measured due to their volatile responses.

The Pilot Stewards were asked to rate how comfortable each horse was during the noseband measurement process. Rankings of comfort were given on a subjective scale of 0–10 with 0 being extremely uncomfortable and uncooperative, and 10 extremely comfortable and cooperative. Just under half (49.0%, *n* = 270) of horses that were measured were ranked as a 10 with ranks of 8 and 9 each having 69 (12.5%) horses. Overall, 83.7% (*n* = 461) of horses that were measured scored a 7 or above on the comfort scale. Thirty-eight horses (6.9%) scored less than 5 on the comfort scale.

While all Stewards who were surveyed unanimously agreed (100%, *n* = 27) that an overtightened noseband poses a welfare issue, most Stewards (62.9%, *n* = 17) believed that tight nosebands are only present in a small subset of riders. Of the Stewards surveyed 59.3% (*n* = 16) felt that the current EC rules were sufficient to address overtight nosebands during competition while 40.7% (*n* = 11) of the Stewards disagreed. Among those who disagreed, those Stewards who participated in the noseband measuring pilot project made up a higher percentage (43.8%, *n* = 7) than those Stewards who did not participate (36.4%, *n* = 4). The Stewards mostly agreed (84%, *n* = 21) that rules surrounding noseband fit should be standardized across all disciplines, but only eight (32%) felt that noseband measurements should be part of mandatory tack checks.

Over 80% of the Pilot Stewards reported that they felt safe performing the noseband measurements. While Stewards felt that noseband measurements could take place before competitors entered the warm-up ring (40.7%, *n* = 11), while in the warm-up ring (40.7%, *n* = 11) or upon exiting the competition ring (51.9%, *n* = 14), they felt that measurements should not be taken just prior to entering the competition ring (66.7%, *n* = 18).

#### 3.1.2. Steward Survey Qualitative Results

Overall the Pilot Stewards were quite supportive of the initiative and never expressed any concerns for their safety. The opportunity to provide education to coaches, trainers and riders and to raise awareness not only within Canada’s equestrian community but worldwide was highlighted.

*I had 3 trainers come to me and ask to be checked by the taper gauge, and there was a very positive response to the whole thing!*

*This is not just national anymore, it has a broader impact improving horse welfare worldwide*

*We could lead the world in this if we are the country that makes it happen. Lots of people are tossing out ways it wouldn’t work, so we should prove that it can*

Most comments were very positive and there were numerous examples of riders proactively asking to be measured and loosening their horse’s noseband of their own volition.

*This rider wanted [his noseband] to be checked at every horse show so that people would see that his horses were fine*

Negative comments revolved around the tool itself. It was felt that the tool was bulky and clumsy to use, and Stewards wondered if a different prototype that was more slender and sleek would make measuring easier. There was also support for a Canadian-made and environmentally sustainable product.

*I would have preferred something smoother so the dirt wouldn’t get caught up and something smoother that would fit in the pocket*

Pilot Stewards found measuring padded nosebands and flash nosebands most problematic. The flash attachment often necessitated a tight noseband and excessive padding interfered with being able to slide the taper gauge under the noseband. Stewards also commented on other issues such as improperly positioned nosebands (too high or too low) and the bridle fit in general. Stewards requested training not only on using the taper gauge itself, but also in how to deal with riders, owners or coaches who are resistant to being measured and take defensive action against difficult messages.

*Stewards need to be educated on how to handle the riders if they find a [nose]band that is too tight using the tool. How to approach it without confrontation*

### 3.2. Member Survey

#### 3.2.1. Member Survey Quantitative Results

Members of Equestrian Canada who responded to the survey (*n* = 1528) trained or competed mostly in hunter or jumper (65.1%, *n* = 996), dressage (50.5%, *n* = 772) or eventing (15.3%, *n* = 234). Survey respondents classified themselves as competitors (45.7%, *n* = 699), amateur riders (45.4%, *n* = 694), coaches (31.5%, *n* = 481), trainers (28.3%, *n* = 432), recreational riders (17.5%, *n* = 268) or professional athletes (15.4%, *n* = 236).

The majority of respondents indicated that their horse always wore a noseband (82.9%, *n* = 1266) and 24 respondents (1.6%) stated their horse only wore a noseband during competitions with 62.5% of these riders (*n* = 15) indicating that they were competing in a discipline that required a noseband. Just over 15% of respondents (15.6%, *n* = 236) noted that their horses did not regularly wear a noseband.

**Figure 4:**
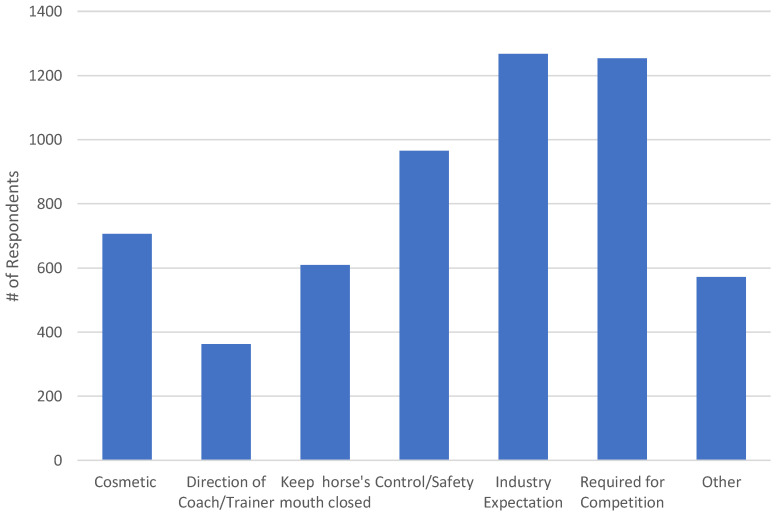
Reasons why member survey respondents (*n* = 1528) use a noseband on their horse. Note that respondents could select multiple reasons.

Across all disciplines, the top four common reasons for using a noseband were that it was an industry expectation, it was a rule requirement by the discipline in which the respondent competed, it was used for control/safety purposes and because it looked good (Figure 4). “Other” reasons for using a noseband included the belief that it improved the contact with the bit, it was used for attaching other equipment (e.g., flash, martingale), that using a bitless bridle or hackamore, which relies on the noseband, was being used, that it was not removable from the bridle or that respondents had never questioned its presence. The role of the respondent influenced the reasons for using a noseband, with professional/high performance riders indicating they used a noseband for control/safety (21.8%, *n* = 100; Χ^2^ = 14.147, *p* < 0.001) and to keep a horse’s mouth closed (16.6%, *n* = 76; Χ^2^ = 24.395, *p* < 0.001) more so than competitors (16.8%, *n* = 240 and 8.8%, *n* = 125 respectively), amateurs (16.5%, *n* = 221 and 9.8%, *n* = 131 respectively), coaches (16.8%, *n* = 157 and 12.3%, *n* = 115 respectively), trainers (16.7%, *n* = 144 and 12.2%, *n* = 105 respectively) or recreational riders (13.9%, *n* = 66 and 6.8%, *n* = 32 respectively). However Cramer’s V interpretation indicated only a weak effect (0.096 and 0.126 respectively).

Almost all respondents agreed that overtightened nosebands presented a welfare issue (87.8%, *n* = 1341) although respondents who had their horse’s noseband measured during the 2021 competition season believed overtightened nosebands were less of an issue (82.8%, *n* = 140; Χ^2^ = 4.285, *p* = 0.038, Cramer’s V = 0.053) than those who did not have a measurement taken (88.4%, *n* = 1201). Similarly, fewer professional/high performance riders felt overtightened nosebands were a welfare issue (76.4%, *n* = 181; Χ^2^ = 33.885, *p* < 0.001, Cramer’s V = 0.149) compared to competitors (90.1%, *n* = 627), amateurs (88.6%, *n* = 613), coaches (86.8%, *n* = 415), trainers (86.1%, *n* = 371) or recreational riders (94.8%, *n* = 254; Figure 4).

**Figure 5:**
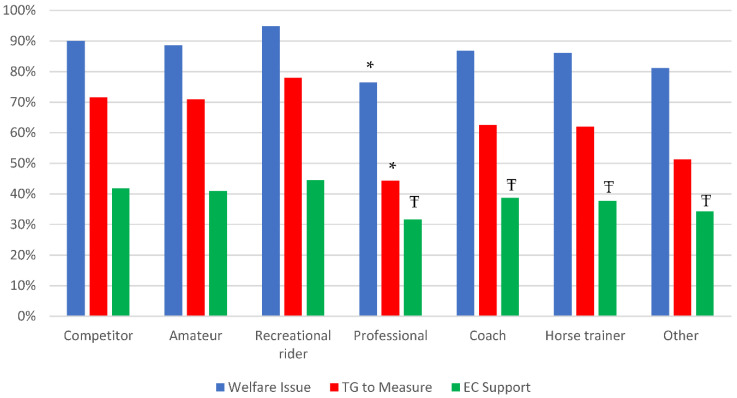
Percentage of member survey respondents (*n* = 1528) who agreed that overtightened nosebands were a welfare issue, that a taper gauge was a fair way to measure noseband fit and who supported Equestrian Canada in advancing horse welfare rules in sport according to the role they represent in the equestrian community (note that respondents could select multiple roles). Differs within role * *p* < 0.0001, Ŧ *p* = 0.009.

Over half the respondents agreed that nosebands should be able to fit two fingers underneath (51.5%, *n* = 787) with 21.3% (*n* = 326) stating that only one finger was acceptable. Respondents mostly agreed that all disciplines should have the same standardized rules for noseband fit (74.4%, *n* = 1137) and that a measurement gauge was a fair method to measure noseband fit (68.0%, *n* = 1039) although slightly fewer respondents agreed that noseband fit should be measured at the frontal nasal plane (65.0%, *n* = 992). Fewer professional/high performance riders (44.3%, *n* = 105; Χ^2^ = 77.281, *p* < 0.001, Cramer’s V = 0.225) felt that a taper gauge was a fair way to measure noseband fit compared to competitors (71.6%, *n* = 498), amateurs (71.0%, *n* = 491), coaches (62.6%, *n* = 299), trainers (61.9%, *n* = 267) or recreational riders (78.0%, *n* = 209; Figure 5). Respondents were divided on whether they felt that measurements for noseband fit should be performed at the Steward’s discretion (45.6%, *n* = 697) or that all nosebands should be checked like other tack at competitions (41.6%, *n* = 636). Only 7.7% (*n* = 117) of respondents believed that measurements for noseband fit should not be included in tack checks.

Respondents (80.6%, *n* = 1231) agreed or strongly agreed to support the role of Equestrian Canada to develop new rules that focus on equine welfare in consultation with subject matter experts although professional/high performance riders (31.6%, *n* = 150), coaches (38.7%, *n* = 370), trainers (37.7%, *n* = 325) and others (34.2%, *n* = 80; Χ^2^ = 17.060, *p* = 0.009, Cramer’s V = 0.106) were less supportive compared to competitors (41.8%, *n* = 582), amateurs (41.0%, *n* = 567) and recreational riders (44.4%, *n* = 238; Figure 5).

#### 3.2.2. Member Survey Qualitative Results

There were 2163 comments submitted across seven different open-ended questions. Most comments on why respondents used a noseband revolved around the understanding that the noseband improved contact with the bit and that it was just part of the uniform.

*Every English bridle I’ve ever owned has had a nose band. I assume there must be a reason!*

There was also awareness that the noseband contributed to horse welfare as it prevented the bit from being pulled through the mouth, reduces oral lesions and allows riders to ride with less rein tension.

*[the noseband] protects the horse’s mouth when properly adjusted*

For many the function of the noseband was to attach other equipment such as a flash, blinkers for driving horses, a nose net, a martingale or it doubled as a halter.

Most of the welfare issues associated with overtightened nosebands pertained to physical effects such as restricted breathing, nerve and tissue damage, bone remodeling, open wounds and inability to swallow. However survey respondents felt that welfare was only compromised in certain situations such as if the noseband was tightened for an extended length of time.

*I feel like it is situation dependent/horse dependent end it is extremely subjective as to what is too tight versus what is appropriate on specific horse for specific reasons*

Similarly respondents most often commented that a two-finger measurement depended on the horse, the type of noseband, the discipline, the horse’s conformation, breed, temperament and size.

*There may be times when the noseband needs to be tighter than usual*

Many voiced the opinion that a “one size fits all” rule was not appropriate.

*Two fingers on a miniature horse or small pony is too much but works well for a draft horse*

*Every horse is different, they’re not all built the same so it’s important that proper noseband fitting is unique to each individual horse*

Many respondents were unsure about 1.5 cm as the ideal measurement for noseband fit, as well as the reasoning behind taking measurements on the frontal plane of the nose compared to other areas of the face (e.g., under the chin or on the side of the nose), particularly when it was outside of their own discipline. There was an echo for using the time-honored methods.

*Anybody should be able to use the old perfectly good method of making sure that 2 fingers can be comfortably inserted between jaw and noseband*

The question of using the taper gauge as a fair measure of noseband fit offered an opportunity for the respondents to comment on their reasoning for being for or against its use. The main reason cited by those who did not agree with the use of the taper gauge was that they did not know what it was or how it was used to measure noseband fit. A concern was raised for the safety of both horse and rider during measurements.

*It seems unsafe, potentially painful, and a biohazard*

Many respondents reiterated their earlier thoughts in the open comments such as making nosebands optional or banning certain types of nosebands, but some new information arose as well. Respondents seemed keen to receive education on noseband fit and measuring.

*Tight nosebands are not only a welfare issue, but an education and training issue*

*It would be great to send out a gauge with membership so owners/coaches/trainers could get their horse used to the process AND so they could accurately check their nosebands at home so there would no surprises at competitions*

There seemed to be a discrepancy between available scientific evidence that supported or identified recommendations for noseband fit and measurement and who to believe.

*There is plenty of research to support that tight nosebands are a welfare concern*

*The origin of the 2 finger rule has no scientific basis so all the “scientific” research is seriously flawed*

*The challenge is who defines who the “experts” are. And who determines whether they are “experts”*

*Veterinary professionals should be the ones deciding the gauge for nose bands and what is safe and what is not safe*

There were also numerous comments opposing the idea of noseband measurements.

*I have not come across ANYONE who is in favor of this proposed rule and am saddened to think that our voices are not being heard*

*Whole discussion about adding another rule on top of already existing rules very sad. I absolutely don’t see the relevance*

*This issue has been over complicated with excess time and resources invested in something most riders have known and been following for years*

Equally there were numerous comments supporting the idea of noseband measurements and the fact that the national equestrian body was dedicating time and effort to this.

*Even though there will be great resistance, I believe it is paramount that this welfare is pushed to the forefront*

*I am proud of EC for taking this first step in this pilot project and going further than the FEI ruling which measures noseband tightness only on the side of the horse’s face*

*This noseband gauge is finally an objective way to measure between horses and by different stewards*

Finally many comments concentrated on the plethora of other issues that are equally as important, if not more important, such as other items of tack, drug use, bullying, footing, poor riding and coaching, lunging and horse slaughter.

## 4. Discussion

Equestrian Canada (EC), the national governing body for equestrian sports, undertook a proactive assessment of noseband fit on horses competing across a variety of disciplines and levels. Tested horses generally had space for the accepted two-finger (1.5 cm) measurement under their noseband with only a small proportion adjusted too tightly to allow measurement by the taper gauge. A similar study on competition horses in Belgium, Ireland and the UK found only 7% of nosebands were loose enough to meet the 1.5 cm measurement [6] while a Dutch study found slightly more than half of horses had passed [24]. Compared to these findings, Canada is on par with Germany where 70% of horses tested met the two-finger measurement [25]. Additionally, EC surveyed both Stewards and members for their thoughts and perceptions related to noseband fit, measurement using a standardized gauge and potential rules around noseband fit. While a majority of survey respondents agreed on certain aspects, other areas reflected distinct differences of opinion. Some survey questions had ambiguous wording, asked about multiple parameters within a single question or provided limited answer choices which may have resulted in a variety of interpretations or misunderstanding from participants. Additionally, Stewards who volunteered to be part of the noseband measuring pilot project presumably did so out of interest and thus results may be confounded by this bias.

Nosebands are not an essential component of the bridle [2] although a majority of the member survey respondents indicated that their horses wore a noseband all the time. Similar to results from other studies [2,12,26] the most common noseband was a cavesson. Industry expectations and discipline norms seem to dictate noseband use although there were a disconcerting number of survey respondents who never questioned the presence of a noseband. A significant motivation for noseband use was the fact that many disciplines require it during competition [3,27,28,29,30]. Professional and high performance riders reported using nosebands more for control and safety reasons, perhaps because they are riding hotter, fitter horses. These results support recent research showing hotter breeds and advanced level performance horses were more apt to wear restrictive nosebands in sales advertisements [26]. Another reason riders used a noseband was cosmetic, a common reason cited by other researchers as well [2]. Certain disciplines and tack are associated with a particular “look” or aesthetic which often includes a noseband. The use of a noseband to attach other equipment such as martingales opens the door to investigation of the need for these items, as martingales may amplify pressures under the noseband [9].

For a noseband to work properly as a riding aid there must be space between the noseband and the nasal plane without restricting the natural behavior and communication abilities of the horse [4]. The noseband should apply pressure when the horse opens their mouth and release the pressure when the mouth closes in the way many other riding cues work with negative reinforcement [2,5,6,31]. As the level of training increases, the pressure of cues should become lighter [32] and restrictive equipment should not be required for the horse to respond to the rein aids. In recent years, alternative styles of nosebands have been designed with an ergonomic approach to reduce pressure on sensitive areas such as the poll and bridge of the nose [33]. However, a tighter noseband increases the horse’s sensitivity to the bit by reducing the ability to move their tongue to dissipate bit pressure [5,6,9,31]. Thus, use of a tighter noseband may increase rider control and safety but, paradoxically, high levels of stress caused by a tight noseband can result in conflict behaviors such as bucking, rearing, shying and bolting [9] which may create more of a safety risk for the rider.

Measures of heart rate, eye temperature and behavior show that stress is present even when the horse simply wears a noseband without working, despite many horses being accustomed to it, and stress levels increase concomitantly with noseband tightness [10,11]. Heart rate response to an overtightened noseband equaled a similar level of stress response that horses had shown to unfamiliar objects [10]. A state of deprivation related to the inability to perform natural behaviors while wearing an overtight noseband has also been identified [10]. Overtightened nosebands may result in physical damage such as lesions at the corners of the lips [12]. Pressures levels exceeding those that cause nerve and blood vessel damage in humans [17], impaired blood flow to the muzzle [11] and bone remodeling [19] are other potential side effects.

Respondents of the Steward and member surveys were widely in agreement that overtightened nosebands were a welfare issue for horses. Most of the Stewards surveyed believed that overtightened nosebands were only an issue with a small subset of riders and not widespread across Canadian competitors. Professional and high performance riders were less convinced that tight nosebands were a welfare issue or that a taper gauge was a fair method to measure noseband fit, perhaps due to the pressure from judge feedback that penalizes horses displaying oral behaviors [21,24]. Some high performance riders may believe that their horse’s nosebands need to be tighter due to the level at which they compete [24]. Tight nosebands can prevent the opening of the mouth along with restriction of other oral behaviors [2,10]. Ideally though, this “submission” should be achieved through training rather than force [32]. Other studies have also noted that overtightened nosebands can cover up a lack of poor training or rider skill [9,11,24].

Riders are commonly taught to be able to fit two fingers underneath the horse’s noseband for correct fit. The origin of this recommendation is not clear, but it has appeared in equestrian texts since 1956 [19] and is largely regarded as the industry standard. Pressure levels tested on the head of an equine cadaver measured an exponential increase under nosebands tightened more than 1.5 finger widths, indicating that the two-finger estimate was undeniably correct [34]. However using finger size as a measurement is highly subjective as finger size varies greatly between individuals and thus yields different levels of tightness of the noseband depending on the person [5]. This led a research team to standardize the traditional two-finger measurement at 1.5 cm [11]. Accordingly, a standardized taper gauge was constructed eliminating the subjectivity of using fingers to measure noseband fit [23]. In previous studies measuring noseband fit at competitions, the same 1.5 cm measurement from the frontal plane of the nose was used [6,12]. Though multiple member survey respondents commented that they preferred measurements to be taken from under the chin or on the side of the face, the 1.5 cm measurement using the TG (or any other form of measurement, including fingers) is intended to be taken from the frontal nasal plane. Softer tissues on the cheek can be depressed to accommodate the gauge allowing higher amounts of pressure to be applied by the noseband while still generating a “passing” measurement, and the site under the chin may sport additional padding that interferes with correct measurements [23]. Some survey respondents expressed concerns regarding the horse having felt pain or discomfort when the TG is “*shoved*” between the noseband and the sensitive, thin skin of the frontal nasal plane. However, the TG is meant to be able to slide to the appropriate stop without force. If force must be applied to insert the gauge, the noseband is too tight and the measurement should stop. Just over half of the members surveyed agreed that this 1.5 cm measurement at the frontal nasal plane was an appropriate guideline for noseband fit across disciplines, but the remainder still need to be convinced.

Despite the plethora of research by leading scientists on the perils of overtightened nosebands [4,5,6,10,11,12,13,19,30] many members either did not know about the research results or were highly suspicious of them. Science skepticism is not a new concept and many factors such as religious, moral and political beliefs along with an understanding of basic science influence how people evaluate and integrate knowledge [35]. It could also be that the equestrian field is steeped in tradition [36] leading to an uphill struggle to influence human behavior change from “what they have always done”. Cognitive dissonance is a phenomenon whereby factual information that is contrary to personal beliefs motivates the belief holder to either deny personal responsibility, trivialize the facts, process only selective information that fits within their belief model, or change their attitude and behavior completely [37]. Since changing attitudes and behavior is the most difficult step, cognitive dissonance more often contributes to inaction, no sense of urgency for action, or selective action that further fosters polarization of the issue. Thompson and Haigh [38] specifically studied the uptake of equitation science research by equestrians and found that those disinclined to believe science may best be persuaded by acknowledging their beliefs without trivializing them while nudging them to think about other approaches. Communicating scientific evidence in a positive manner that fits with existing rider beliefs—i.e., the horse’s welfare is a top priority—and using veterinarians as an information conduit may be the best path for changing attitudes [39,40].

Rules regarding noseband fit are currently discipline-specific and often vague. FEI Showjumping rules state “the Grand Jury has the right, based on veterinary advice, to forbid the use of a bit or noseband that may cause injury to the Horse” [27]. FEI Driving and Para-Driving rules state “Any nosebands, attachments or ancillary equipment which impede or are likely to impede the free intake of air into the nostrils of the Horse are not permitted” [28]. These rules are mirrored by EC in Driving and Para-Driving, Hunter, Jumper, Equitation and Hack Rules for National Jumper Divisions [41,42]. The FEI Eventing rules make no mention of noseband tightness or potential misuse at all [29] while the EC Eventing Rules state that “the noseband may never be so tightly fixed as to harm the horse” [43]. The EC Dressage and Para-Dressage Rules have the most comprehensive rules stating “A horse’s noseband must not be over tightened. It must be possible to place at least one finger between the horse’s cheek and the noseband” [44]. The dressage rules also regard tight nosebands as dubious equipment that a steward may check at any time [44]. In EC’s general rules “nosebands used in such a way that they interfere with the horse’s breathing or be tight enough to cause pain or discomfort” are considered an act of cruelty that is not tolerated [22]. The Breed Sports, General Performance, Western, Equitation, Endurance, Reining and Para-Reining and Vaulting rulebooks do not make any mention of noseband tightness or fit at all [45,46,47,48,49]. This may be a result of nosebands not often used in many of these disciplines. Another comment that appeared repeatedly was a request for nosebands to be made optional in all disciplines with some survey respondents stating that a well-trained horse should not need a noseband: “If dressage is [a] test of training, [then] horses at high levels should not require a noseband at all”.

Both the members and Stewards surveyed mostly agreed that noseband fit rules should be standardized across all disciplines. However open comments underscored the idea that noseband fit depended on many factors including the individual horse, the breed, the conformation, the rider, the type of noseband, the discipline and the circumstances. Indeed, many Western disciplines do not include the use of a noseband at all, making a standardized rule about nosebands senseless. While the noseband taper gauge admittedly cannot account for all types of head conformation, it is within the realm of science to create a gauge that could [23]. For now though, a standardized rule across the board may not be well received by competitors who already feel weighted down by rules. Sentiment leaned toward using a standardized measure only in warranted situations such as a tie-breaker, but not to make it mandatory for all competitors.

Safety of the Steward, rider/handler and horse was another common concern mentioned in the survey comments. Prior to the start of the noseband measuring pilot project EC provided training material to their Stewards on the correct use of the TG including information on how Stewards can keep themselves and everyone involved with the measurement safe. All measurements occurred when the rider was dismounted. While there were numerous member comments regarding fearful responses from their own horses when confronted with the TG, the Stewards themselves did not voice concerns over safety and only a small percentage of horses were rated as uncomfortable with the procedure. Nevertheless, the TG should be introduced quietly and carefully to the horse being measured, and riders themselves have an onus to ensure their horses are comfortable with the procedure as part of the showing environment. Stewards did not recommend taking the noseband measurement just before the rider enters the competition ring. While this would likely be the best way of ensuring that the horse is not competing with an overtightened noseband, a horse who is aroused may chew or tense their jaw which could affect the accuracy of the measurement by temporarily creating less space between the noseband and the frontal nasal plane [23]. Measuring before the horse has begun to work or after exiting the competition ring was suggested as a better time as the horse is more relaxed then. There were concerns raised over biosecurity when using the TG on multiple horses at competitions. However the plastic device can easily be disinfected by submersion in a dilute alcohol solution. Having multiple devices on hand would allow one to be in use while another is being disinfected. The tool itself was commented on as being bulky and clumsy to use, but other noseband gauges have been designed and the suggestion for a tool made from sustainable material is worth investigating.

A strong level of support toward welfare-focused developments in Canadian equestrian sport indicates that the survey respondents have a keen desire to do right by their horses. This agrees with findings from Clayton and Williams [2] who found that riders place tremendous value on their horse’s comfort (and by extension, their welfare) when making noseband-related decisions. Such a high degree of support for welfare-focused developments indicates forward-thinking in Canadian equestrians to improve the quality of life for horses and ensure a good public image and a future for equestrian sport. This is not surprising, as there is almost unanimous agreement and awareness among Canadians that welfare issues are present within their equine industry [50]. However, despite recognition of welfare issues, Canadian survey respondents were divided on how best to address these issues and which groups of horses may be most at risk of welfare challenges [50]. Similarly both Steward and member survey respondents in this study demonstrated opposing viewpoints on appropriate noseband fit, the use of a standardized tool and the implementation of noseband measures across disciplines. A comparable study of Dutch equestrians found similar contrasting beliefs surrounding noseband measures and horse welfare [24]. Additionally, member survey respondents cited numerous other issues they felt were more important to address than noseband fit, such as banning specific types of nosebands, horse abuse and poor training techniques. Nevertheless, these comments all pertained to a concern for horse welfare in general.

Lack of knowledge is a strong underlying force behind welfare issues in the equine industry [51]. It is evident from the member survey comments regarding the perceived lack of research on noseband fit that a gap exists between current evidence-based data and end-user knowledge uptake. Numerous member survey respondents indicated that they did not know what the noseband taper gauge was or how it was used, similar to another survey where less than a third of respondents were able to identify a device used to measure noseband tightness when shown a photo of it [2]. Equestrians regularly fail to recognize signs of distress in their horses or misinterpret behavioral cues [52] indicating a lack of understanding of horse behavior. However the introduction of noseband measurements may be a great opportunity to advance equestrian practice through educational outreach attached to the issue of noseband fit. Stewards commented on the interest and thirst for knowledge that competitors displayed when approached to participate in the noseband measuring pilot. Stewards themselves, once properly trained, could be a conduit for imparting current information on noseband fit. It would be an easy step to include information on noseband fit within coach accreditation requirements or a mandatory short informative video as part of competitor licensing. Other countries such as Switzerland already have similar mandates in place where horse owners are required to obtain an equine welfare certificate before caring for multiple horses [53]. Successful communication and uptake of equitation science by equestrians allows continual improvement of horse quality of life and welfare through the implementation of research results.

## 5. Conclusions

The noseband measuring pilot project undertaken by volunteer Stewards over the 2021 competition season found that most horses had nosebands fastened appropriately to fit a standardized measure of one- or two-fingers between the frontal plane and the noseband. Only 10% of competitor horses had their nosebands so tight that the TG could not slide underneath. Both Steward and member survey respondents agreed that overtightened nosebands were a welfare issue for horses. While in theory member survey respondents agreed with a standardized measure of noseband fit across disciplines, copious comments indicated differences of opinions on how, when and why noseband measures should take place. In particular, professional and high performance riders were less convinced of the fairness of using a taper gauge and less supportive of new rule implementations. Misinformation or misunderstanding of research results was evident, indicating a need for educating riders, coaches and trainers on the importance of appropriate noseband fit and the consequences of overtightened nosebands. Making nosebands optional in disciplines that currently require them was suggested. Despite differing viewpoints, there was a clear desire to improve equine quality of life.

## Data Availability

Data can be made available on request.

## Supplementary Materials

The following supporting information can be downloaded at: https://www.mdpi.com/article/10.3390/ani12192685/s1, Supplement S1: Pilot Steward survey; Supplement S2: All Steward survey; Supplement S3: EC Member survey; Supplement S4: Noseband types.

## References

[1] Chechushkov I.V., Usmanova E.R., Kosintsev P.A. (2020). Early evidence for horse utilization in the Eurasian steppes and the case of the Novoil’inovskiy 2 Cemetery in Kazakhstan. J. Archaeol. Sci. Rep..

[2] Clayton H.M., Williams J.M. (2022). Know your noseband: An exploration of the factors that influence riders’ choice of noseband. J. Vet. Behav..

[3] Holmes T.Q., Brown A.F. (2022). Champing at the Bit for Improvements: A Review of Equine Welfare in Equestrian Sports in the United Kingdom. Animals.

[4] McGreevy P.D., Doherty O., Channon W., Kyrklund K., Webster J. (2017). The use of nosebands in equitation and the merits of an international equestrian welfare and safety committee: A commentary. Vet. J..

[5] Casey V., McGreevy P.D., O’Muiris E., Doherty O. (2013). A preliminary report on estimating the pressures exerted by a crank noseband in the horse. J. Vet. Behav..

[6] Doherty O., Casey V., McGreevy P., Arkins S., Munderloh U.G. (2017). Noseband Use in Equestrian Sports—An International Study. PLoS ONE.

[7] Hill E., McGreevy P.D., Caspar G., White P., McLean A.N. (2015). Apparatus use in popular equestrian disciplines in Australia. J. Vet. Behav..

[8] Hawson L.A., McLean A.N., McGreevy P.D. (2010). The roles of equine ethology and applied learning theory in horse-related human injuries. J. Vet. Behav..

[9] Condon V.M., McGreevy P.D., McLean A.N., Williams J.M., Randle H. (2022). Associations between commonly used apparatus and conflict behaviors reported in the ridden horse in Australia. J. Vet. Behav..

[10] Fenner K., Yoon S., White P., Starling M., McGreevy P.D., Munderloh U.G. (2016). The effect of noseband tightening on horses’ behavior, eye temperature, and cardiac responses. PLoS ONE.

[11] McGreevy P., Warren-Smith A., Guisard Y. (2012). The effect of double bridles and jaw-clamping crank nosebands on temperature of eyes and facial skin of horses. J. Vet. Behav..

[12] Uldahl M., Clayton H.M. (2018). Lesions associated with the use of bits, nosebands, spurs, and whips in Danish completion horses. Eq. Vet. J..

[13] Dyson S., Bondi A., Routh J., Pollard D., Preston T., McConnell C., Kydd J.H. (2021). An investigation of behavior during tacking-up and mounting in ridden sports and leisure horses. Equine Vet. Educ..

[14] Dyce K.M., Sack W.O., Wensing C.J.G. (2010). Textbook of Veterinary Anatomy.

[15] Doherty O.M. (2016). An Investigation into the Oro-Nasal Pressures Used in the Control of the Ridden Horse. Ph.D. Thesis.

[16] Robinson N., Bye T.L. (2021). Noseband and poll pressures underneath bitted and bitless bridles and the effects on equine locomotion. J. Vet. Behav..

[17] Rydevik B., Lundborg G. (1977). Permeability of intraneural microvessels and perineurium following acute, graded experimental nerve compression. Scand. J. Plast. Reconstr. Surg..

[18] Crago F., Shea G., James O., Schemann K., McGreevy P.D. (2019). An opportunistic pilot study of radiographs of equine nasal bones at the usual site of nosebands. J. Vet. Behav..

[19] Perez-Manrique L., Leon-Perez K., Zamora-Sanchez E., Davies S., Ober C., Wilson B., McGreevy P.D. (2020). Prevalence and distribution of lesions in the nasal bones and mandibles of a sample of 144 riding horses. Animals.

[20] Federation Equestre Internationale. 2022. Dressage Rules. 25th Edition. Switzerland. https://inside.fei.org/sites/default/files/FEI_Dressage_Rules_2022_Clean_Version_V2.pdf..

[21] Hawson L.A., McLean A.N., McGreevy P.D. (2010). Variability of scores in the 2008 Olympic dressage competition and implications for horse training and welfare. J. Vet. Behav..

[22] Equestrian Canada. 2022. Equestrian Canada Rules. Section A: General Regulations. https://www.equestrian.ca/cdn/storage/resources_v2/KFbHDPDHmMbfFnkqH/original/KFbHDPDHmMbfFnkqH.pdf..

[23] Doherty O., Conway T., Conway R., Murray G., Casey V. (2017). An Objective Measure of Noseband Tightness and Its Measurement Using a Novel Digital Tightness Gauge. PLoS ONE.

[24] Visser E.K., Kuypers M.M.F., Stam J.S.M., Riedstra B. (2019). Practice of noseband use and intentions towards behavioral change in Dutch equestrians. Animals.

[25] Pahl D., Kienapfel K. Noseband tightness on National German leisure competition in low and medium classes. Proceedings of the International Conference Equitation Science.

[26] Merkies K., Copelin C., McPhedran C., McGreevy P. (2022). The presence of various tack and equipment in sale horse advertisements in Australia and North America. J. Vet. Behav..

[27] ederation Equestre Internationale. 2022. Jumping Rules. 27th Edition. Switzerland. https://inside.fei.org/sites/default/files/Jumping_Rules_2022_final_clean.pdf..

[28] Federation Equestre Internationale. 2022. Driving Rules. 12th Edition. Switzerland. https://inside.fei.org/sites/default/files/FEI%20-%20Driving%20Rules%202022_Clean_v6.pdf..

[29] Federation Equestre Internationale. 2022. Eventing Rules. 25th Edition. Switzerland. https://inside.fei.org/sites/default/files/2022%20Eventin%20Rules_clean%20version.pdf..

[30] Weller D., Franklin S., Shea G., White P., Fenner K., Wilson B., Wilkins C., McGreevy P. (2020). The reported use of noseband in racing and equestrian pursuits. Animals.

[31] Manfredi J., Clayton H.M., Derksen F.J. (2005). Effects of different bits and bridles on frequency of induced swallowing in cantering horses. Equine Comp. Exerc. Physiol..

[32] Ödberg F.O., Bouissou M.-F. (1999). The development of equestrianism from the baroque period to the present day and its consequences for the welfare of horses. Equine Vet. J..

[33] Murray R., Guire R., Fisher M., Fairfax V. (2015). A bridle designed to avoid peak pressure locations under the headpiece and noseband is associated with more uniform pressure and increased carpal and tarsal flexion, compared with the horse’s usual bridle. J. Equine Vet. Sci..

[34] Doherty O., Casey V., Conway R. Changes in pressures exerted on sub-noseband tissues by tightening the noseband. Proceedings of the International Society for Equitation Science Annual Conference.

[35] Rutjens B.T., Heine S.J., Sutton R.M., van Harreveld F. (2017). Attitudes towards science. Adv. Exper. Soc. Psych..

[36] van Weeren P.R. (2008). How long will equestrian traditionalism resist science?. Vet. J..

[37] Moser D., Steiglechner P., Schlueter A. (2022). Facing global environmental change: The role of culturally embedded cognitive biases. Environ. Dev..

[38] Thompson K., Haigh L. (2018). Perceptions of Equitation Science revealed in an online forum: Improving equine health and welfare by communicating science to equestrians and equestrian to scientists. J. Vet. Behav..

[39] Lofgren E.A., Rice B.M.G., Brady C.M. (2022). Exploring perceptions of equine welfare scenarios using a positive approach. J. Appl. Anim. Welf. Sci..

[40] Pickering P., Hockenhull J. (2020). Optimising the efficacy of equine welfare communications: Do equine stakeholders differ in their information-seeking behavior and communication preferences?. Animals.

[41] Equestrian Canada. 2022. Equestrian Canada Rules. Section C: Driving and Para-Driving. https://www.equestrian.ca/cdn/storage/resources_v2/weWWorQJQrhHBQ4JG/original/weWWorQJQrhHBQ4JG.pdf..

[42] Equestrian Canada. 2022. Equestrian Canada Rules. Section G: Hunter, Jumper, Equitation and Hack. https://www.equestrian.ca/cdn/storage/resources_v2/zrrGFNTpA9Rz7fKgQ/original/zrrGFNTpA9Rz7fKgQ.pdf..

[43] Equestrian Canada. 2022. Equestrian Canada Rules. Section D: Eventing. https://www.equestrian.ca/cdn/storage/resources_v2/wgnbtpwpechHQzeGL/original/wgnbtpwpechHQzeGL.pdf..

[44] Equestrian Canada. 2022. Equestrian Canada Rules. Section E: Dressage and Para-Dressage Article. https://www.equestrian.ca/cdn/storage/resources_v2/bezEJo3wHP9JNRt7X/original/bezEJo3wHP9JNRt7X.pdf..

[45] Equestrian Canada. 2022. Equestrian Canada Rules. Section B: Breeds. https://www.equestrian.ca/cdn/storage/resources_v2/6xcouH26ApK94KJdF/original/6xcouH26ApK94KJdF.pdf..

[46] Equestrian Canada. 2022. Equestrian Canada Rules. Section F: General Performance, Western, Equitation. https://www.equestrian.ca/cdn/storage/resources_v2/2cMTieom5F92adRHY/original/2cMTieom5F92adRHY.pdf..

[47] Equestrian Canada. 2022. Equestrian Canada Rules. Section J: Endurance. https://www.equestrian.ca/cdn/storage/resources_v2/KncCrHfFih8f4Hju9/original/KncCrHfFih8f4Hju9.pdf..

[48] Equestrian Canada. 2022. Equestrian Canada Rules. Section K: Reining and Para-Reining. https://www.equestrian.ca/cdn/storage/resources_v2/32Ccob58jAR2YPHFG/original/32Ccob58jAR2YPHFG.pdf..

[49] Equestrian Canada. 2022. Equestrian Canada Rules. Section K: Vaulting. https://www.equestrian.ca/cdn/storage/resources_v2/Dv5btcF56s9j2jddR/original/Dv5btcF56s9j2jddR.pdf..

[50] DuBois C., Nakonechny L., Derisoud E., Merkies K. (2018). Examining Canadian equine industry participants’ perceptions of horses and their welfare. Animals.

[51] DuBois C., Hambly-Odame H., Haley D.B., Merkies K. (2018). An exploration of industry expert perception of Canadian equine welfare using a modified Delphi technique. PLoS ONE.

[52] Bell C., Rogers S., Taylor J., Busby D. (2019). Improving the recognition of equine affective states. Animals.

[53] Lesté-Lasserre C. (2015). A Look at Switzerland’s Equine Protection Laws. The Horse.

