# Supplementary material for: Noseband Fit: Measurements and Perceptions of Canadian Equestrians
Source: Animals (Basel). 2022 Oct 6;12(19):2685. doi: 10.3390/ani12192685 (PMC9559623; doi:10.3390/ani12192685)
Supplement: Supplementary file 1 [file animals-12-02685-s001.zip › S1 Pilot Steward Survey.pdf]

Pilot Steward Perceptions Survey Questions and Answer Options

1. How many events/competitions did you attend as a steward during the duration of the pilot? (pilot events)
    - Less than 5
    - Between 5-10
    - More than 10
2. Which disciplines were the pilot events you conducted measurements at? Check all that apply
    - Eventing
    - Jumper
    - Hunter
    - Dressage
    - Breed Sports
    - Eventing (cross country)
    - Other – Write In (Required)
3. What level of competition were your pilot events? (check all that apply)
    - Bronze (or eventing Equivalent)
    - Silver
    - Gold
    - FEI
    - Other – Write In (Required)
4. On average, how many measurements did you complete during each event?
    - 1-5
    - 5-10
    - 10-30
    - 30+
5. Did you feel safe conducting measurements with the ISES measuring gauge provided to you for the pilot?
    - Yes, I did
    - I require more training to feel more comfortable
    - I did not try because I did not feel safe
    - Other – Write In (Required)
6. What was rider/trainer acceptability like for being measured when asked?
    - Slider on a scale from “would not participate” to “asked to be measured”

7. In general, how comfortable were horses with the measurement process?
    - Slider on a scale from “could not be measured” to “very easy to measure”
8. Do you feel we have a noseband tightness issue within the Equestrian Canada field of play?
    - Yes, and I feel it is important to address
    - I do, in a small portion of our riders
    - I do not feel there is a noseband issue and feel the current rules are sufficient
    - Prefer not to answer
9. Do you feel the current Equestrian Canada rules support you in addressing overtightened nosebands during your role as a steward?
    - Yes
    - No
10. If there were to be a rule to standardize the measurement of noseband tightness with a tool (which tool is still in consideration) in all disciplines, which of the following statements best represents your view in the implementation?
    - There is no need for a standardized tool for measuring noseband tightness, or alignment of the rules across the disciplines
    - The rules should be aligned across all disciplines to one measurement, and there should be compulsory checks like the other tack checks (i.e., boot check)
    - The rules across disciplines should be aligned to one measurement for noseband tightness, and a tool can be used if needed or as a “tie breaker”, but not a compulsory check for all horses
    - Other: Please state
11. What is important for stewards to implement/practice when approaching a horse and rider to measure/discuss a noseband’s tightness?
    - Open comment box
12. What elements/areas of the competition setting do you recommend for engaging with horses and riders for measurements?
    - Before they enter the warmup ring
    - In the warmup ring
    - Exiting the warmup ring
    - Before entering the competition ring
    - Exiting the competition ring
    - Other: Please Describe

13. What elements/areas of the competition setting do you *not recommend* engaging with horses and riders for measurements?
    - Before they enter the warmup ring
    - In the warmup ring
    - Exiting the warmup ring
    - Before entering the competition ring
    - Exiting the competition ring
    - Other: Please Describe
14. What did you enjoy about participating in the noseband pilot project?
    - Open comment box
15. What did you dislike about participating in the noseband pilot project?
    - Open comment box
16. What information would you like to share with Equestrian Canada about the steps forward?
    - Open comment box
17. What education and training should be focused on by Equestrian Canada for competitors?
    - Open comment box
18. What education and training should be focused on for Equestrian Canada stewards/TDs?
    - Open comment box
19. In what areas do you feel we need further research?
    - Open comment box
20. Is there any other information you would like to share with us?
    - Open comment box
