# Supplementary material for: Noseband Fit: Measurements and Perceptions of Canadian Equestrians
Source: Animals (Basel). 2022 Oct 6;12(19):2685. doi: 10.3390/ani12192685 (PMC9559623; doi:10.3390/ani12192685)
Supplement: Supplementary file 1 [file animals-12-02685-s001.zip › S2 All Steward Survey.pdf]

All Steward Perceptions Survey Questions and Answer Options

1. How many events/competitions did you attend as a steward? (pilot events)
    - o Less than 5
    - o Between 5-10
    - o More than 10
    - o More than 20
2. Which disciplines do you mainly work as a steward or technical delegate? (check all that apply)
    - o Eventing
    - o Jumper
    - o Hunter
    - o Dressage
    - o Breed Sports
    - o Eventing (cross country)
    - o Other – Write In (Required)
3. What level of competition do you mainly act as a steward/technical delegate? (check all that apply)
    - o Bronze (or eventing Equivalent)
    - o Silver
    - o Gold
    - o FEI
    - o Other – Write In (Required)
4. On average, how many measurements did you complete during each event?
    - o 1-5
    - o 5-10
    - o 10-30
    - o 30+
5. Do you feel that overtightened nosebands are a horse welfare issue?
    - o Yes
    - o No
6. Do you feel we have a noseband tightness issue within the Equestrian Canada field of play?
    - o Yes, and I feel it is important to address
    - o I do, in a small portion of our riders
    - o I do not feel there is a noseband issue and feel the current rules are sufficient
    - o Prefer not to answer

7. Do you feel the current Equestrian Canada rules support you in addressing overtightened nosebands during your role as a steward?
    - Yes
    - No
8. If there were to be a rule to standardize the measurement of noseband tightness with a tool (which tool is still in consideration) in all disciplines, which of the following statements best represents your view in the implementation?
    - There is no need for a standardized tool for measuring noseband tightness, or alignment of the rules across the disciplines
    - The rules should be aligned across all disciplines to one measurement, and there should be compulsory checks like the other tack checks (i.e., boot check)
    - The rules across disciplines should be aligned to one measurement for noseband tightness, and a tool can be used if needed or as a "tie breaker", but not a compulsory check for all horses
    - Other: Please state
9. What is important for stewards to implement/practice when approaching a horse and rider to measure/discuss a noseband's tightness?
    - Open comment box
10. What elements/areas of the competition setting do you recommend for engaging with horses and riders for measurements?
    - Before they enter the warmup ring
    - In the warmup ring
    - Exiting the warmup ring
    - Before entering the competition ring
    - Exiting the competition ring
    - Other: Please Describe
11. What elements/areas of the competition setting do you *not recommend* engaging with horses and riders for measurements?
    - Before they enter the warmup ring
    - In the warmup ring
    - Exiting the warmup ring
    - Before entering the competition ring
    - Exiting the competition ring
    - Other: Please Describe

12. What information would you like to share with Equestrian Canada about the steps forward?
    - Open comment box
13. What education and training should be focused on by Equestrian Canada for competitors?
    - Open comment box
14. What education and training should be focused on for Equestrian Canada stewards/TDs?
    - Open comment box
15. In what areas do you feel we need further research?
    - Open comment box
16. Is there any other information you would like to share with us?
    - Open comment box
