# Supplementary material for: Noseband Fit: Measurements and Perceptions of Canadian Equestrians
Source: Animals (Basel). 2022 Oct 6;12(19):2685. doi: 10.3390/ani12192685 (PMC9559623; doi:10.3390/ani12192685)
Supplement: Supplementary file 1 [file animals-12-02685-s001.zip › S3 Member Survey.pdf]

Sport License Holder Survey Questions and Answer Options

1. What discipline(s) do you typically compete or train in?
    - Dressage
    - Show Jumping
    - Hunter/Jumper
    - Breed Sports
    - Endurance
    - Driving
    - Vaulting
    - Eventing
    - Other (please specify)
2. How do you classify yourself (check all that apply)?
    - Competitor/Student
    - Amateur
    - Recreational Rider
    - Professional/High Performance Rider
    - Coach
    - Horse Trainer
    - Other (please specify)
3. Does your horse(s) always wear a noseband?
    - Yes
    - No
    - Only at competitions
4. Why does your horse wear a noseband?
    - None of the above
    - Cosmetic (it looks good)
    - Direction of coach/trainer
    - To help keep the horse’s mouth closed
    - Control/Safety
    - Industry Expectation/Discipline Norm
    - The discipline(s) I compete in require it in their rules
    - Other (please specify)

5. Did you have your noseband measured during this past summer’s noseband pilot project?
    - Yes
    - No
6. Do you feel overtightened nosebands are a horse welfare issue?
    - Yes
    - No
    - *Comment box attached for additional input*
7. Do you think that all disciplines should have the same standardized noseband tightness rules?
    - Yes
    - No
    - *Comment box attached for additional input*
8. Do you feel a measurement gauge is fair way to measure noseband tightness during Equestrian Canada Competition?
    - Yes
    - No
    - Other (please specify)
9. Do you agree noseband tightness should be measured at the frontal nasal plane?
    - Yes
    - No
    - Other (please specify)
10. Do you feel noseband tightness checks should be a mandatory tack check, or at random/discretion of the steward?
    - Nosebands should be measured like other tack
    - The steward should be able to measure nosebands that look tight or at random
    - There should be a minimum percentage (example 10%) of horses measured at each competition
    - None of the above
11. Do you feel the gauge should be used to perform all noseband checks, or only in 'tie breaker' scenarios?
    - The gauge should be used in all noseband checks
    - The EC steward should be able use their fingers or gauge at their discretions
    - The gauge should be used when there is discrepancy between the competitor and the steward
    - The gauge should not be used in noseband checks

12. Do you agree that 2 fingers (1.5cm) at the frontal nasal plane should be the tightness a noseband can be in all disciplines?
    - Yes
    - No
    - 1 finger is sufficient
    - None of the above
    - *Comment box attached for additional input*
13. Do you agree with following statement: "Equestrian Canada should work with subject matter experts to be a world leader in advancing horse welfare rules in Equestrian Sport?
    - Strongly agree
    - Agree
    - Neither agree nor disagree
    - Disagree
    - Strongly Disagree
14. Do you have any other information or comments you would like to share?
    - Open comment box
