# Supplementary material for: Noseband Fit: Measurements and Perceptions of Canadian Equestrians
Source: Animals (Basel). 2022 Oct 6;12(19):2685. doi: 10.3390/ani12192685 (PMC9559623; doi:10.3390/ani12192685)

Supplement S4: Various types of nosebands encountered by Stewards when measuring noseband fit on horses at equestrian competitions in Canada.

Cavesson	Noseband with flash	Crank noseband

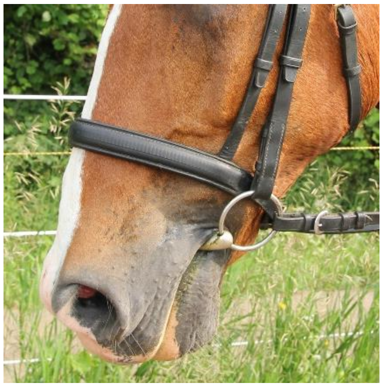
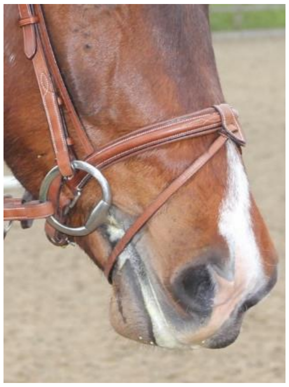
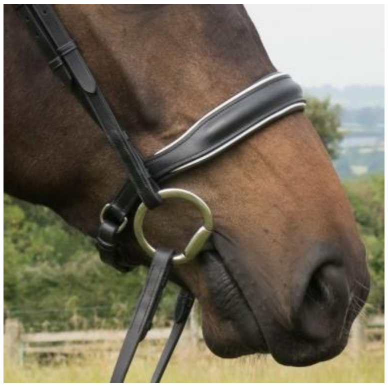

Figure 8 noseband	Ergonomic noseband (Schockemöhle style)	Drop noseband

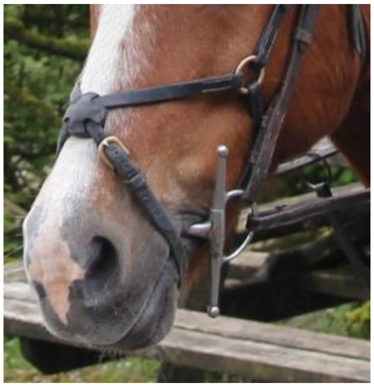
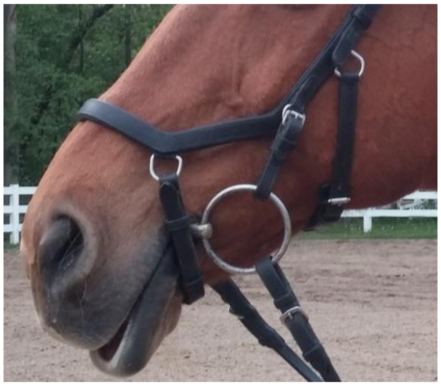
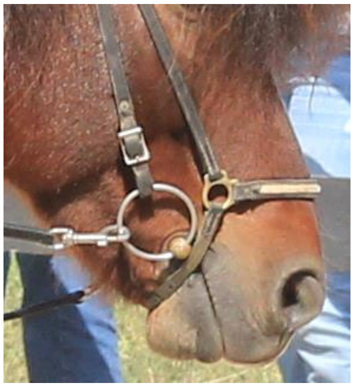

Mechanical hackamore

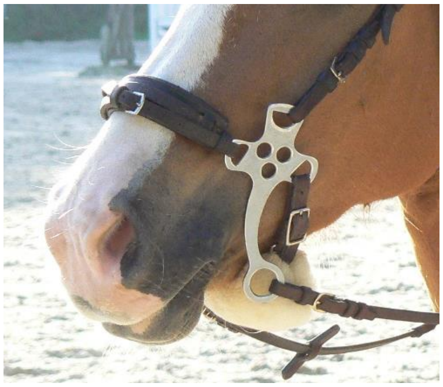

Supplement: Supplementary file 1 [file animals-12-02685-s001.zip › Supplement S4 - noseband types.pdf]
